# Risk factors associated with acquiring gastrointestinal infections in UK international travellers: a case–control study

**DOI:** 10.1017/S0950268826101058

**Published:** 2026-01-22

**Authors:** Nicola K Love, Yanshi Yanshi, Parisha Katwa, Iman Mohamed, Dipti Patel, Hilary Kirkbride, Sooria Balasegaram

**Affiliations:** 1 UK Health Security Agency North East (UKHSA), UK; 2 University of Liverpool, UK; 3 National Travel Health Network and Centre (NaTHNaC), UK

**Keywords:** bacillary, case–control studies risk factors, cryptosporidiosis, dysentery, giardiasis, *Salmonella* infections, travel

## Abstract

International travel is thought to be a major risk factor for developing gastrointestinal illness in England. Transmission is thought to be more likely in countries which have lower food hygiene standards, poorer sanitation, and lack of access to clean water. However, many studies are conducted within travel clinic settings which may bias findings. Here, we present a case–control study undertaken in returning English travellers in the community conducted with cases of gastrointestinal illness notified to UKHSA.

All Cryptosporidiosis, Giardiasis, non-typhoidal Salmonellosis, and Shigellosis cases notified to the UK Health Security Agency (UKHSA) between 01 July 2023 and 15 October 2023 were asked to complete an anonymous electronic questionnaire if travelling during their incubation period. Asymptomatic travellers were recruited as controls via a market research panel and asked to complete the same questionnaire. A destination water, hygiene, and sanitation score were derived from the WHO ‘Attributable fraction of diarrhoea to inadequate WASH’ dataset. Demographics, travel details, and exposures while travelling were compared by Pearson’s chi-squared test, and pathogen and destination specific multivariable analyses were performed using a forward stepwise approach.

A total of 653 cases and 483 controls were included. The odds of being a case were significantly higher when travelling to countries outside of the EU (OR:4.6, 95%CI:3.5–6.0; p = <0.001) and to countries with high-risk WASH score (OR 6.6, 95%CI:4.9–9.1; p = <0.001), particularly Egypt, Mexico, Tunisia, and Turkey. For those travelling to a low-risk destination, eating undercooked meat or fish and swallowing water from environmental water sources were significantly associated with higher odds of illness by multivariable analysis (p < 0.05). At high-risk destinations, eating foods consumed on excursions, swallowing water from environmental sources, and eating foods from hotel buffets were significantly associated with higher odds of being a case.

Travel to popular tourist destinations is a potentially under-recognized risk factor for acquiring gastrointestinal infections. Exposures at low-risk destinations were broadly similar to risk factors in the UK. Exposures in high-risk destinations highlighted potential risks associated with catered hotels and tourist excursions which should be explored further.

## Introduction

With the exception of years impacted by the COVID-19 pandemic (2020-2022), international travel undertaken by UK residents has seen a year-on-year increase from 58.3 million visits in 2001 to 93.1 million in 2019, with an estimated 86.2 million visits made in 2023 [1]. European destinations – particularly Spain, France, Italy, and Greece – continue to be the most popular travel destinations for UK residents. However, around 15 million visits were made to countries outside of Europe and North America in 2023 [1] with globalization leading to increased travel to locations in low- and middle-income countries (LMICs). Such destinations are often associated with increased health risks as a result of endemic and vector-borne diseases [2].

Gastrointestinal illnesses remain one of the most common health complaints reported by travellers. Often referred to as travellers’ diarrhoea, the attack rate is estimated to range from 20% to 60% with morbidity believed to be the highest travellers returning from LMICs where water, sanitation, and hygiene may be compromised [3, 4]. Evidence from one English region suggested that international travel contributed considerably to the burden of gastrointestinal morbidity with around half of Hepatitis A, *Shigella* and non-typhoidal *Salmonella* infections, a third of giardia and Cryptosporidium infections and 20% of O157 E.coli infections in the region diagnosed in individuals who had travelled internationally during their incubation period [5].

The risk of developing gastrointestinal illness while travelling is often thought to be associated with travel to destinations such as Southeast Asia and Africa and activities including backpacking and visiting friends and relatives (VFR; [4, 6, 7]). However, many studies are conducted within travel clinic settings, which may introduce bias towards certain types of travel, specific destinations, and more severe infections. Having a better understanding of travel-associated enteric infections could help to improve pre-travel advice and support public health actions, which could ultimately lead to a reduction in reported gastrointestinal infections in England. Here, we present a case–control study undertaken in returning English travellers in the community conducted with cases who were diagnosed with a notifiable gastrointestinal infection to better understand the risks associated with acquiring a gastrointestinal infection while travelling internationally in the general population.

## Methods

### Case identification and recruitment

Laboratory-confirmed cases of *Cryptosporidium, Giardia, Salmonella* (non-typhoidal), and *Shigella* notified to the UK Health Security Agency (UKHSA) from English laboratories as part of UK statutory health protection regulations [8] were identified in UKHSA’s case management system HPZone for 8 of the 9 UKHSA regions. In the ninth region, where routine exposure questionnaires are performed for the four pathogens of interest, line lists were provided indicating cases with travel during their incubation period. Cases were excluded if their record did not have a valid mobile phone number or email address. However, a pilot of London cases without telephone or email details were contacted by letter to improve uptake, as there were more London cases without valid contact details. No sampling frame or sample size was applied as it was not known how many cases in eight of the nine English regions would have travelled.

A link to a study website containing participant information and the questionnaire was disseminated by text or email to all cases notified between 01 July and 15 October 2023 using the UK Government Notify messaging system. The study date range was chosen to coincide with the start of the summer holiday period in England. Messages were sent out twice weekly to newly reported cases. Symptomatic individuals were asked to complete the electronic questionnaire if they had travelled internationally in the 7 days prior to symptom onset if diagnosed with *Salmonella* or *Shigella*, and in the 14 days prior to onset if diagnosed with *Cryptosporidium* or *Giardia.* Responses were received between 01 July 2023 and 04 November 2023.

### Control recruitment

Controls were recruited using a for-profit market research company between 28 September 2023 and 02 October 2023, as described previously [9]. Controls received financial compensation for responses. One response per person was permitted with no sampling frame applied. Quotas were not used to select participants. However, for responses on behalf of children, the questionnaire was specifically directed to a subset of panel members known to have children with the wording of the questionnaire changed to reflect interest in responses on behalf of children. Controls were recruited with the aim of an overall ratio of one control per case. Screening questions were used to select eligible participants (UK resident, international travel after 01 July 2023 and no symptoms of gastrointestinal illness during travel or in the 14 days after travelling).

### Data collection

Cases and controls were asked to complete an electronic questionnaire developed and hosted in Snap Survey (Snap 11 Professional; Bristol, UK). The questionnaire contained closed and open-ended questions capturing information on symptoms and health-seeking behaviours (cases only), travel destination and type of travel, exposures while travelling, access to travel health advice, and awareness of travel health advice. Informed consent was provided by respondents prior to questionnaire completion. No personal identifiable information was obtained apart from postcode; however, cases were provided with an identifier which would allow for linkage to surveillance data held by UKHSA.

### Data analysis

All analyses were performed in R Studio (R version 4.2.0, Rstudio version 2024.04.1 + 748). Case and control responses were excluded from the analysis if not meeting date or travel eligibility criteria. Scores for water, sanitation, and hygiene (WASH) were derived using the World Health Organization (WHO) ‘Attributable fraction of diarrhoea to inadequate water, sanitation and hygiene’ dataset [10]. Countries with a score of ≤0.18 were defined as low-risk and > 0.18 were defined as high-risk WASH score countries (Supplementary Table S1 **)**. For the purposes of the WASH analysis, scores for Maderia and the Azores were derived from Portugal, the Canary Islands were derived from Spain, Cyprus and Northern Cyprus were both reported as Cyprus, and Saint Kitts and Nevis was derived from neighbouring Antigua and Barbuda. Classification into EU and non-EU destinations and geographical areas was based on the UK Office for National Statistics (ONS) International Passenger Survey Travelpac dataset [11]. Demographic characteristics, destination of travel and exposures while travelling were compared using Pearson’s Chi-squared test with Fisher’s exact test used where appropriate using the *tbl_summary* from the *gtsummary* package. Multivariable analysis was performed using *tbl_uvregression* from the *gtsummary* package using the glm method. Final models added variables using a forward stepwise approach including all variables with a p value <0.2 and a case attack rate of 10% for all analyses apart from the pathogen-specific Shigella analysis where a 20% case attack rate was used. All graphs were produced in *ggplot2.*

## Results

Between 01 July 2023 and 15 October 2023, 7,654 laboratory confirmed cases of *Cryptosporidium* (2,710), *Giardia* (1,291), non-typhoidal *Salmonella* (3,046), and *Shigella* (607) were notified to the UK Health Security Agency from English laboratories, of which 5,730 cases had valid contact details and were sent the questionnaire (*Cryptosporidium* n = 1,410; *Giardia* n = 1,069; *Salmonella* n = 2,711, and *Shigella* n = 540). From a previous study [5], it was estimated that 43% of cases over the period were likely to be travel associated (42% for *Cryptosporidium*, 31% for *Giardia*, 48% for *Salmonella*, and 45% for *Shigella*) based on the proportion of cases reporting travel in one English region over the same time period [5]. 689 Travelbug questionnaire responses were received, giving an estimated response rate of 28% for cases estimated to be travel related (34% *Cryptosporidium*, 28% *Giardia*, 25% *Salmonella*, and 20% *Shigella*; Supplementary Figure S1). Nine cases reporting symptom onset prior to or on the day of travel, 15 cases who reported illness more than 14 days after returning to the UK, and 12 cases reporting travel and onset prior to 01 June 2023 were excluded. Overall, 653 cases were included in the study (188 *Cryptosporidium* (29%), 83 *Giardia* (13%), 308 *Salmonella* (47%), and 48 *Shigella* (7%), and 26 who did not provide a specific pathogen (4%)), responses were proportionate to the number of cases contacted with each pathogen.

### Case symptoms and health-seeking behaviours

Self-reported onset dates ranged from 03 June 2023 to 03 October 2023 (week 22 - week 40 2023) for the 653 included cases. Reports were highest between 14 August 2023 and 3 September 2023. Diarrhoea was reported by 98% of respondents (n = 640), with abdominal pain (n = 572; 88%), nausea (n = 451; 69%), and vomiting (n = 252; 39%) less commonly described (Table 1). Vomiting was most frequently associated with *Cryptosporidium* infection (47%; n = 89/252), while fever was more common in those diagnosed with *Salmonella* (55%) and *Shigella* (56%). Bloody diarrhoea was reported by 19% of cases (n = 121); commonly by those diagnosed with *Salmonella* and *Shigella* (26% and 25%, respectively), although a small number of *Cryptosporidium* (8.0%) and *Giardia* cases (9%) also reported this symptom. Gastrointestinal symptom duration was reported for 435 cases (67%) who had reported resolution of gastrointestinal symptoms at the time of questionnaire completion, with a mean duration of diarrhoea and vomiting of 11.4 days reported (SD:7.2; *Cryptosporidium* 9.2 days, *Giardia* 16.6 days, *Salmonella* 10.5 days, and *Shigella* 14.1 days). A total of 280 cases reported having at least one day of absence from employment or education due to symptoms (58% of those who responded, n = 483), with an average duration of absence of 6.5 days (SD: 8.3 days) reported. Gastrointestinal illness in co-travellers was reported by 42% of cases (n = 276), with 182 reporting co-travellers with onset prior to their symptoms and 132 reporting co-travellers with onset after onset of their illness. A total of 119 cases reported illness in other guests at their accommodation, of which a third (n = 40) reported evidence of illness in communal areas. Reports of illness in communal areas was most commonly by those diagnosed with *Cryptosporidium.*

**Table 1.** Clinical details of English residents diagnosed with cryptosporidium, Giardia, non-typhoidal Salmonella, and Shigella who self-reported international travel during their incubation period and responded to the electronic questionnaire (n = 653)

		Overall	Pathogen specific			
	Symptom	N = 653	Cryptosporidium, N = 188	Giardia, N = 83	Salmonella, N = 308	Shigella, N = 48
Symptoms^a^	Diarrhoea	640 (98%)	184 (98%)	80 (96%)	304 (99%)	46 (96%)
	Blood in stool	121 (19%)	15 (8%)	7 (8.4%)	81 (26%)	12 (25%)
	Abdominal pain	572 (88%)	168 (89%)	65 (78%)	276 (90%)	41 (85%)
	Nausea	451 (69%)	130 (69%)	58 (70%)	217 (70%)	31 (65%)
	Vomiting	252 (39%)	89 (47%)	29 (35%)	106 (34%)	17 (35%)
	Fever	288 (44%)	66 (35%)	15 (18%)	170 (55%)	27 (56%)
	Joint pain	282 (43%)	78 (41%)	23 (28%)	150 (49%)	21 (44%)
	Headache	377 (58%)	106 (56%)	33 (40%)	201 (65%)	25 (52%)
	Dizzy	221 (34%)	65 (35%)	26 (31%)	109 (35%)	15 (31%)
	Tiredness	559 (86%)	156 (83%)	70 (84%)	272 (88%)	41 (85%)
	Other Symptom	67 (10%)	19 (10%)	6 (7%)	38 (12%)	4 (8.3%)
Diarrhoea and vomiting duration (days)^b^,^,c^		11.4 (7.2)	9.2 (3.7)	16.6 (12.6)	10.5 (4.7)	14.1 (9.9)
Number of days hospitalized (in UK)^b^,^d^		3.7 (4.3)	2.4 (2.1)	-	4.2 (5.1)	5.0 (1.4)
Number of days hospitalized (non-UK)^b^,^c^		2.0 (1.6)	-	-	-	-
Antibiotics (% acquired in UK)		234 (86%)	15 (93.3%)	73 (92%)	114 (85%)	21 (84%)
Number of days absent^b^,^,e^		6.5 (8.3)	5.3 (4.1)	9.6 (19.7)	6.4 (4.1)	8.9 (12.1)
High-risk WASH destination (% pathogen total)^f^		288 (49%)	28 (16%)	36 (51%)	176 (64%)	35 (85%)

a n (%).

b Mean (SD).

c Duration of illness missing for 218 cases (70 cryptosporidium, 34 giardia, 92 salmonella and 12 shigella cases).

d Hospitalization duration in UK missing for 22 cases reporting hospitalization. UK hospitalization reported for 18 cryptosporidium (7 missing) and 41 salmonella (9 missing) cases, Hospitalization duration outside UK missing for 2 cases, breakdown by pathogen not shown due to small numbers.

e Employment absence data missing for 376 cases (109 cryptosporidium, 49 giardia, 167 salmonella and 35 shigella cases).

f WASH score missing for 65 cases (9 cryptosporidium, 13 giardia, 32 salmonella and 7 shigella cases).

All cases sought healthcare for their illness in the UK, with most using General practitioners (83%; n = 542) or NHS 111 (23%; n = 153; Supplementary Table S2). A total of 89 cases reported seeking healthcare for their illness while outside of the UK (14%), with pharmacies the most common place to seek advice (n = 46/89; 52%). An identical proportion of cases seeking healthcare while abroad and in the UK were hospitalized (13%; n = 12 outside UK vs. n = 87 within UK), with the average duration of hospitalization of 3.71 days for those hospitalized in the UK (SD:4.27; n = 82 responses) and 2.0 days for those hospitalized while outside the UK (SD:1.60; n = 10 responses; Table 1). Antibiotic consumption was self-reported by 225 cases (34%); 199 cases consumed antibiotics in the UK (88%), 19 outside of the UK (8%), and 7 cases reported taking antibiotics acquired both in the UK and while abroad (3%; Supplementary Table 3). Antibiotics were most frequently taken by *Giardia* cases (n = 71; 76%), with around a third of *Salmonella* and *Shigella* cases also reporting antibiotic use (*Salmonella*: 34% and *Shigella*: 39%; Table 1 and Supplementary Table S3). Cases also commonly reported taking pain medication (n = 334; 51%), antidiarrheals (n = 211; 32%), rehydration salts (n = 250; 38%), and over the counter IBS medications, predominantly hyoscine butylbromide (n = 51; 8%; Supplementary Table S3).

### Demographics of cases and controls

Exposure questionnaires were completed by 629 controls recruited via a commercial market research company. A total of 483 controls were eligible for inclusion (0.7 controls per case) with 146 questionnaires excluded (119 controls reported travel outside of the study period, 11 reported UK travel only, 4 had not travelled, 12 questionnaires were incomplete).

Cases were significantly younger than controls (p = <0.001; 23% vs. 43% over the age of 50 years); however, the proportion of children under 19 years was comparable between the two groups (28% vs. 23%; Supplementary Table S4). Cases were slightly more likely to be female (63% vs. 57%; p = 0.06) and to have been resident in the UK since birth (90% vs. 84%; p = 0.003), although there was no significant difference in ethnicity between the two groups (p = 0.4). Co-morbidities were reported by both cases and controls. Cases were more likely to have inflammatory bowel disease (p = <0.001), while controls were more likely to have diabetes (p = 0.05) and to be immunosuppressed (p = <0.001). Occupations were broadly similar between the two groups, but cases were more likely than controls to be employed in health and social care (11% vs. 7%; p = 0.03).

### Location and type of travel

**Figure 1.**
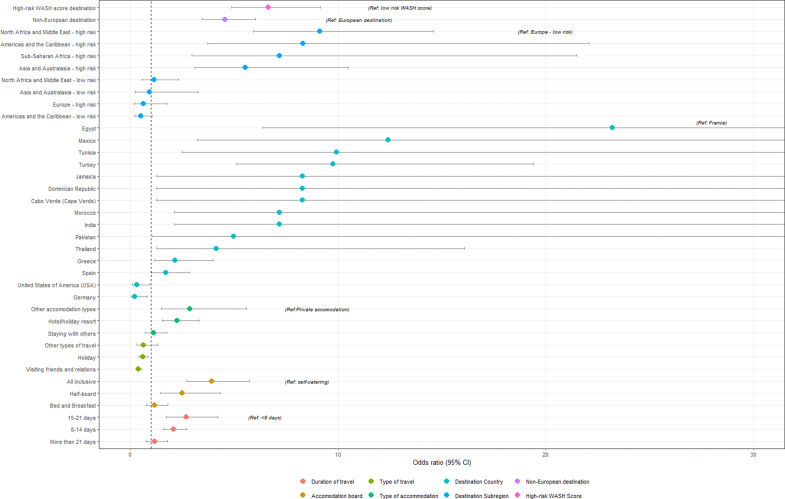
Univariate analysis showing odds ratios and 95% confidence intervals associated with specific travel destinations and forms of travel. Sub-region missing for 68 cases and 1 control. 67 cases missing classification (Europe vs. non-Europe), 65 cases missing WASH score. Accommodation type missing for 73 cases. Accommodation board was a conditional question, therefore missing for 133 controls and 190 cases. Only countries with >4 visitors included in country analysis (see Supplementary Table 1 for details). Only those with p = <0.06 included in figure. p = 0.059 for Jamaica, Cape Verde and Dominican Republic and p = 0.058 for Pakistan; large error bars due to small numbers. For type of accommodation, private accommodation was defined as holiday villa or holiday apartment. ‘Staying with others’ included Airbnb’s where staying with the host, B&B accommodation, hostels, guesthouses and staying with friends of family. ‘Other’ accommodation included camping. Hotels and holiday resorts included cruise holidays. For type of travel, visiting friends and family includes visiting own home abroad. Holiday included both package and independent travel.

Where country of travel was known (588/653 cases; 90% and 100% controls), cases were significantly more likely to have travelled to countries outside of the EU (47% vs. 19%; OR: 4.6, 95%CI: 3.5–6.0; p = <0.001). Irrespective of geographical area, travel to countries with poorer water, sanitation and hygiene (WASH) as indicated by a high-risk WASH score was associated with increased odds of being a case (OR 6.6, 95%CI: 4.9–9.2; p = <0.001). The odds of developing a gastrointestinal infection were significantly higher in individuals who had travelled to high-risk WASH score areas in North Africa and the Middle East (OR 9.1, 95%CI: 5.9–14.6; p = <0.001), the Americas and Caribbean (OR 8.3, 95%CI: 3.7–22.1; p = <0.001), Sub-Saharan Africa (OR 7.2, 95%CI: 3.0–21.5; p = <0.001), and Asia and Australasia (OR 5.5, 95%CI: 3.1–10.5; p = <0.001), when compared with low-risk European destinations (Figure 1). Thirteen destinations were associated with higher odds of becoming a case, of which the highest odds were reported for Egypt, Mexico, Tunisia, and Turkey, with the odds of illness in travellers to Egypt 23 times higher than those visiting France. However, odds could not be estimated for all destinations, including for certain high risk countries in Africa and Asia, due to small numbers (See Supplementary Table S1).

Where travel destination was known, 49% of cases travelled to a high-risk WASH destination compared with 13% of controls. Where destination was known, cases diagnosed with Shigellosis, Salmonellosis, and Giardiasis were most likely to have travelled to a high-risk WASH score destination (85%, 64%, and 50%, respectively; Table 1). Cryptosporidiosis was more commonly associated with travel to low-risk destinations (84%).

For both groups, package holidays were the most common type of travel reported (n = 368 cases and n = 271 controls; 56% for both). The majority of cases (n = 411; 70.5%) and controls (n = 276; 57.1%) stayed in a hotel or holiday resort while travelling. Staying in hotels or holiday resorts was associated with higher odds of being a case (OR: 2.3, 95%CI: 1.5–3.3; p = <0.001), when compared with staying in private accommodation. The odds of illness were also significantly higher in those who had stayed in all-inclusive accommodation (OR: 3.9, 95% CI:2.7–5.7; p = <0.001) or half-board accommodation (OR; 2.5, 95%CI:1.4–4.3; p = 0.001), when compared with self-catering accommodation. For the 13 destinations associated with higher odds of being a case, six (Egypt, Tunisia, Turkey, Dominican Republic, Cabo Verde) had more than 80% of cases on all-inclusive package holidays (Table 2).

**Table 2.** Countries identified in univariate analysis as being associated with higher odds of illness (compared with reference destination of France) indicating the number of travellers reporting package holiday travel and all-inclusive package holiday travel to destination

		Controls	Cases		
	OR (95% CI)	Total reporting destination (% package travellers)	Total reporting destination	Total reporting package holiday travel	Total reporting all-inclusive package holiday travel
Egypt	23.2 (6.4–150)	2 (100%)	28	26 (93%)	25 (89%)
Mexico	12.4 (3.2–82.1)	2 (100%)	15	10 (67%)	10 (67%)
Tunisia	9.9 (2.5–66.5)	2 (100%)	12	11 (92%)	11 (92%)
Turkey	9.8 (2.5–66.5)	18 (83%)	106	96 (91%)	87 (82%)
Jamaica	8.3 (1.3–162)	1 (0%)	5	5 (100%)	5 (100%)
Dominican Republic	8.3 (1.3–162)	1 (100%)	5	5 (100%)	4 (80%)
Cabo (Cape) Verde	8.3 (1.3–162)	1 (0%)	5	5 (100%)	5 (100%)
Morrocco	7.2 (2.1–33.1)	3 (33%)	13	9 (69%)	5 (38%)
India	7.2 (2.1–33.1)	3 (67%)	13	0 (0%)	0 (0%)
Pakistan	5.0 (1.1–35.3)	2 (50%)	6	2 (33%)	1 (17%)
Thailand	4.1 (1.3–16.1)	4 (25%)	10	2 (20%)	0 (0%)
Greece	2.2 (1.1–4.0)	37 (81%)	48	38 (79%)	23 (48%)
Spain	1.7 (1.0–2.9)	109 (69%)	112	74 (66%)	47 (42%)

*Note:* Only countries with >4 visitors included in country analysis (see Supplementary Table S1 for details). Associated with higher odds (p = <0.06); p = 0.059 for Jamaica, Cape Verde and Dominican Republic and *p* = 0.058 for Pakistan, all others *p* = <0.05.

### Exposures while travelling

In a multivariable analysis, exposures differed by high- and low-risk destinations (Table 3). For those travelling to low-risk destinations, eating undercooked meat or fish, eating meat or fish purchased from local restaurants and airports, drinking purified water, and swallowing water from environmental water sources (rivers, lakes, sea, and swimming pools) were all found to be associated with higher odds of illness. In high-risk destinations, eating foods consumed on trips or excursions, swallowing water from environmental sources, drinking fruit juice or smoothies, and eating foods from hotel buffets were all associated with higher odds of being a case.

**Table 3.** Risk exposures significantly associated with higher odds of being at case by mulitvariable analysis for low-risk WASH destinations and high-risk WASH destinations

		Case % exposed	Control % exposed	Univariate			MVA		
				OR	95%CI	P value	OR	95%CI	P value
Low-risk travel	Eating undercooked meat/fish	10%	5%	2.2	1.2–3.9	0.01	4.5	1.9–11.1	<0.001
	Drinking purified water	20%	7%	3.4	2.1–5.5	0.001	4.4	2.1–9.6	<0.001
	Swallowing water from environmental sources	47%	17%	4.0	2.8–5.8	0.001	3.1	1.9–5.2	<0.001
	Swimming in rivers and lakes	10%	4%	3.0	1.6–5.9	0.001	15.2	2.4–111	0.005
	Travel to EU destinations	93%	91%	0.4	0.2–0.5	0.001	5.1	1.1–25.2	0.04
	Consuming uncooked vegetables^a^	57%	53%	1.2	0.9–1.6	0.2	2.1	1.0–4.1	0.04
High-risk travel	Swallowing water from environmental sources	34%	13%	3.5	1.7–8.2	0.002	87.6	11.8–1 –190	<0.001
	Consuming fruit juices or smoothies	45%	33%	1.7	1.0–3.1	0.07	21.6	4.39–156	<0.001
	Consuming fruit on trips and excursions	10%	2%	6.7	1.4–121	0.06	3,373	21.5–234,889	0.01
	Consuming uncooked vegetables on trips & excursions^a^	14%	5%	3.0	1.1–12.8	0.07	192	4.4–17,498	0.01
	Consuming uncooked vegetables^a^	65%	75%	0.6	0.3–1.1	0.13	107	4.2–5,107	0.01
	Consuming fruit from a local restaurant	18%	26%	0.6	0.3–1.2	0.2	19.7	2.0–274	0.02
	Consuming dairy products^b^ from a local restaurant	13%	21%	0.6	0.3–1.2	0.11	18.7	2.0–262	0.02
	Consuming pre-cut fruit^c^	39%	28%	1.7	0.9–3.1	0.11	6.5	1.6–34.0	0.02
	Any food consumed from hotel buffets	60%	38%	2.5	1.4–4.4	0.002	63.7	2.4–4,595	0.03
	Consuming eggs	72%	64%	1.4	0.8–2.5	0.2	5.7	1.2–32.6	0.04
	Meat or fish consumed at an airport	10%	5%	2.2	0.7–9.3	0.2	49.3	2.1–5,947	0.05
	‘Other’^d^ foods consumed from hotel buffets	53%	26%	3.2	1.8–6.1	0.001	64.9	1.0–5,864	0.06
	Consuming meat or fish on trips and excursions	14%	2%	9.4	2.0–168	0.03	142	1.3–71,645	0.07

*Note:* Only includes those exposures for which attack rate in cases >10%.

a Uncooked vegetables includes raw salad leaves, raw salad vegetables, uncooked or unpeeled vegetables and fresh herbs.

b Dairy products include milk, yoghurt, cheese or ice cream.

c Fruit includes unpeeled fruit or fruit washed in tap water, fruit peeled or washed in bottled water, pre-cut fresh fruit and fruit juices and smoothies.

d Other foods includes eggs, cakes and pastries, pre-prepared sandwiches, barbecued food, rice, dips, ice lollies and curried dishes.

Pathogen-specific exposures were determined (Table 4) by multivariable analysis. For Cryptosporidium, swimming in rivers and lakes (OR: 11.8, 95% CI: 1.97–88.0, P = 0.01), swimming in swimming pools where there was evidence of faeces or vomit, and drinking purified water were all found to be significantly associated with higher odds of being a case. Visiting a petting farm or zoo was also identified as a potential exposure (p = 0.07). Like Cryptosporidium, Giardia infections were also associated with water contact, with contact with river or lake water and drinking purified or untreated water associated with higher odds of being a case (p = <0.05). Giardia was also significantly associated with visiting a high-risk destination in the Americas or Caribbean (OR: 48.3, 95% CI: 1.47–2069, p = 0.03). For Salmonella infections, visiting high-risk WASH destinations, particularly those in Asia or Australasia, consuming meat or fish on trips or excursions, consuming certain foods from hotel buffets and staying in catered accommodation were all associated with higher odds of illness. Visiting high-risk WASH destinations was also associated with higher odds of illness in those with Shigella (OR: 8542, 95% CI:236, 2,027206, p = <0.00).

**Table 4.** Pathogen specific risk exposures significantly associated with higher odds of being at case by mulitvariable analysis

		Case % exposed	Control % exposed	Univariate			MVA		
				OR	95%CI	P value	OR	95%CI	P value
Cryptosporidium	Drinking purified water	19%	7%	3.05	1.84, 5.03	0.001	3.29	1.43, 7.69	0.005
	Swimming in rivers and lakes	15%	4%	3.87	2.14, 7.08	0.001	11.8	1.97, 88.0	0.01
	Evidence of faeces or vomit in swimming pools	9%^h^	0%	24	6.79, 152	0.001	11.3	2.13, 93.8	0.01
	Meat or fish consumed at an airport	15%	5%	3.68	2.05, 6.68	0.001	5.64	1.42, 24.3	0.02
	Consuming meat or fish from a local restaurant	35%	45%	0.65	0.45, 0.92	0.015	2.85	1.09, 7.80	0.04
	‘Other’^a^ foods consumed at an airport	10%	6%	1.73	0.92, 3.18	0.083	4.28	0.91, 20.0	0.06
	Visiting a petting farm or zoo	11%	3%	4.03	2.06, 8.04	0.001	2.66	0.91, 7.85	0.07
Giardia	High-risk destination in Americas or Caribbean	10%	1%	8.52	2.88, 26.5	0.001	48.3	1.47, 2,069	0.03
	Contact with river/lake water (other)^b^	17%	5%	4.27	2.05, 8.67	0.001	27.5	1.42, 713	0.04
	Drinking untreated water	10%	1%	8.52	2.88, 26.5	0.001	24.2	3.30, 197	0.002
	Consuming meat or fish on trips and excursions	10%	3%	3.87	1.49, 9.51	0.004	21.4	2.60, 184	0.004
	Swimming in rivers or lakes	20%	4%	5.69	2.83, 11.3	0.001	18.7	1.01, 468	0.06
	Consuming fruit^c^ you prepared	17%	25%	0.6	0.32, 1.08	0.1	16.4	1.22, 434	0.06
	Consuming dairy^d^ you prepared	13%	20%	0.62	0.30, 1.17	0.2	12.7	1.55, 123	0.02
	Consuming vegetables^e^ purchased from an airport	19%	8%	2.66	1.38, 4.93	0.003	11.9	1.42, 147	0.03
	Drinking purified water	22%	7%	3.56	1.87, 6.59	0.001	5.4	1.34, 22.1	0.02
Salmonella	Consuming meat or fish on trips and excursions	10%	3%	3.63	1.89, 7.35	0.001	18	3.14, 114	0.002
	Consuming vegetables^e^ from ‘other locations’^f^	57%	68%	0.65	0.48, 0.87	0.004	10.8	2.32, 57.5	0.003
	‘Other’^a^ foods consumed from hotel buffets	46%	21%	3.34	2.44, 4.58	0.001	5.06	1.29, 21.5	0.02
	Drinking purified water	12%	7%	1.76	1.08, 2.86	0.023	3.67	1.67, 8.15	0.001
	Visiting a high-risk WASH destination	57%	13%	12.2	8.55, 17.7	0.001	3.62	1.13, 12.1	0.03
	Visiting high-risk destination in Asia or Australasia	11%	3%	4.31	2.33, 8.42	0.001	3.21	0.89, 12.0	0.08
	Undertaking water sports in the sea	12%	7%	1.87	1.14, 3.10	0.014	3.16	1.21, 8.45	0.02
	Swimming in the sea	50%	39%	1.57	1.17, 2.09	0.002	2.31	1.10, 5.00	0.03
	Staying in catered accommodation	56%	29%	3.16	2.35, 4.27	0.001	2.03	1.02, 4.07	0.04
Shigella	Visiting a high-risk WASH destination	73%	13%	40.5	17.5, 111	0.001	8,542	236, 2,027206	<0.001
	Consuming vegetables^e^ from a local restaurant	27%	43%	0.49	0.24, 0.93	0.035	175	3.75, 22,638	0.02
	Consuming food^g^ purchased from an airport	25%	17%	1.64	0.79, 3.20	0.2	14.7	1.73, 255	0.03
	Pre-cut fresh fruit	42%	29%	1.74	0.94, 3.18	0.073	13.2	1.54, 180	0.03

*Note:* Only includes those exposures for which attack rate in cases >10% for cryptosporidium, giardia and salmonella, and attack rates in cases >20% for shigella due to small numbers. Ratio of cases to controls differed by pathogen: Cryptosporidium (2.5 controls per case), Giardia (5.8 controls per case), Salmonella (1.6 controls per case) and Shigella (10.1 controls per case).

a Other foods includes eggs, cakes and pastries, pre-prepared sandwiches, barbecued food, rice, dips, ice lollies, and curried dishes.

b Contact with river/lake water (other) is contact other than swimming or paddling.

c Fruit includes unpeeled fruit or fruit washed in tap water, fruit peeled or washed in bottled water, pre-cut fresh fruit, and fruit juices and smoothies.

d Dairy products include milk, yoghurt, cheese or ice cream.

e Vegetables includes raw salad leaves, raw salad vegetables, uncooked or unpeeled vegetables, and fresh herbs.

f Other locations includes a location other than food you have prepared yourself, food prepared by a host, food from a local restaurant, food from a buffet at a hotel, food from a restaurant, food from another hotel, food stalls, markets, street food, or from an airport. Participants were not asked what the ‘other’ location was.

g Foods include Vegetables^e^, Fruit^c^, meat, and fish, Dairy products^d^ and other^a^ food items.

h Attack rate < 10% as question only asked to those reporting swimming pool exposure.

## Discussion

Here, we present the findings of a case–control study undertaken in returning travellers in the community diagnosed with a notifiable gastrointestinal infection. We show that the odds of being a case are higher in individuals who had travelled outside of the EU and to higher-risk areas which may have poorer water, sanitation, and hygiene. While our study did show higher odds of being a case in travellers who reported to travel to countries or regions which were documented in other studies and in travel guidance to be ‘high risk’ for travel associated GI infections [4, 12, 13], it also highlighted potentially under recognized risks in travel to popular short-haul destinations including Egypt, Tunisia and Turkey. A key strength of this study is that it was conducted in returning travellers identified in the general population, rather than individuals accessing specific travel health services which may introduce bias towards certain countries or types of travel, particularly destinations where travel vaccination or chemoprophylaxis measures are required. Furthermore, the inclusion of cases diagnosed with specific gastrointestinal pathogens allows for the identification of pathogen specific exposures, which could support efforts to reduce the burden of infections in England.

A limitation of this study was that only those infections diagnosed following return to England were included, which may lead to an underestimation of infections, particularly those that may be short-lived or less severe [14]. Likewise, as only a small number of included cases reported seeking care outside of the UK, this study likely excludes individuals who have only sought treatment while travelling and have not visited a healthcare provider when returning to the UK. This is important when considering the context of antimicrobial resistance and the importation of resistant enteric pathogens to the UK [15, 16]. Demographic differences between cases and controls are also an important limitation of this work. Cases were younger than controls, although the proportion of children in both groups was comparable, and were more likely to have been resident in the UK since birth, although there were no differences in ethnicity between the groups. Cases were also more likely to have inflammatory bowel disease and therefore may have been more susceptible to gastrointestinal infections while travelling [17] or may have been more likely to access testing. The study was also conducted over the UK summer holiday period, and travel patterns during this period may not reflect travel at other times of the year.

As reported previously, travel to low- and middle-income countries with high-risk WASH scores was found to be a significant exposure associated with developing a gastrointestinal infection, with destinations in Southern Asian and Africa commonly associated with a higher morbidity [4, 13]. Due to small numbers, it was not possible to determine destination specific odds for all destinations, particularly those in Africa and Asia. However, for destinations reported by more than three participants, several locations were found to be associated with increased odds of illness that were less commonly recognized as being associated with higher morbidity from travel associated gastrointestinal illness. These included several short-haul destinations including Egypt, Tunisia and Turkey where cases had almost exclusively travelled on all-inclusive package holidays. As destinations popular with UK travellers [11], it is not unexpected that a substantial proportion of cases reported travel to these destinations. However, our study suggests that a disproportionate number of gastrointestinal infections may be arising from travel to these locations, with increased odds of illness not observed in travellers to other popular tourist destinations such as Spain, Portugal, or France. In a previous study in residents in North East England diagnosed with notifiable gastrointestinal infections, these destinations were also shown to be associated with higher rates of illness per 100,000 visits [5]. Furthermore, Egypt and Turkey were also identified in the North East study as being commonly associated with clusters of cases linked to hotels or resorts [5]. While in our study, we did not look at commonalities between hotels, just under half of cases reported similar symptoms in a co-traveller and a fifth of cases reported other guests staying in their accommodation were unwell – including reports of illness within communal areas. This suggests that that the true burden of illness associated with international travel may be substantially underestimated, and that clustering of cases within resorts or hotels may be under-recognized. This should be investigated further, particularly as we show that catered accommodation was significantly associated with being a case in our univariable analysis and that eating foods from hotel buffets was associated with illness in our multivariable analysis.

The bacterial and parasitic pathogens included in this study are commonly associated with foodborne and waterborne routes of transmission [18]. Here we show that for travel to low-risk WASH countries, which were predominantly European destinations and frequently Cryptosporidium infections, exposures associated with higher odds of being a case were comparable to known UK exposures for these pathogens – particularly consuming undercooked meat or fish, visiting a petting farm or zoo and swallowing water from environmental water sources [18]. This suggests that risks are likely to be very similar to the UK and therefore more general advice applicable to both UK and destinations may be required. Exposures associated with high-risk destinations were associated with food exposures, including eating foods on trips or excursions and from hotel buffets, and swallowing water from environmental sources. Travel to high-risk destinations was a risk factor for Salmonella, Shigella, and Giardia infections, although a lower number of Giardia and Shigella cases were included in the study.

This work suggests that more needs to be done to raise awareness of risk in those that visit short-haul destinations in low- and middle-income countries where WASH may not be equivalent to the UK and similar European destinations. While this study identifies exposures associated with travel to high-risk destinations, most cases and controls included in the study travelled to low-WASH areas making it difficult to draw conclusions for some risk factors such as destinations due to low numbers. Further studies specifically focusing on travel to high-risk destinations are needed, particularly those that explore exposures and perception of risk in those travelling to popular short-haul destination to help inform public health messages aimed at prevention and reduction of travel associated gastrointestinal illness in travellers and further raise awareness in the travel industry.

## Supporting information

10.1017/S0950268826101058.sm001 Love et al. supplementary material Love et al. supplementary material

## Supplementary material

The supplementary material for this article can be found at http://doi.org/10.1017/S0950268826101058.
