# Supplementary material for: Risk factors associated with acquiring gastrointestinal infections in UK international travellers: a case–control study
Source: Epidemiol Infect. 2026 Jan 22;154:e36. doi: 10.1017/S0950268826101058 (PMC13100926; doi:10.1017/S0950268826101058)
Supplement: Love et al. supplementary material [file S0950268826101058sup001.docx]

**Supplementary** **Table 1 – Destination countries reported by study participants by Water, Sanitation and Hygiene (WASH) score, where high-risk indicates a country with a higher attributable fraction of diarrhea to inadequate WASH.**

|  | **High risk WASH score destination (n=44)** | **Low risk WASH score destination  (n=39)** |
| --- | --- | --- |
| **Americas and the Caribbean** | *Bolivia*  *Colombia*  *Costa Rica*  Cuba  Dominican Republic  Jamaica  Mexico  *Peru* | *Antigua and Barbuda*  *Barbados*  Canada  *Saint Kitts and Nevis*  United States of America (USA) |
| **Asia and Australasia** | *Afghanistan*  *Bangladesh*  *Cambodia*  China  India  Indonesia  *Malaysia*  *Maldives*  *Nepal*  Pakistan  *Philippines*  *Sri Lanka*  *Tajikistan*  Thailand  *Vietnam* | Australia  *Japan*  *Singapore* |
| **Europe** | *Albania*  Bulgaria  *Montenegro*  Romania | Austria  *Azores (Portugal)*  Belgium  Canary Islands (Spain)  Croatia  Cyprus, Northern  Cyprus, Republic of  Czechia  Denmark  France  Germany  Greece  Hungary  Iceland  Ireland  Italy  *Latvia*  *Lithuania*  Madeira (Portugal)  Malta  Netherlands  Norway  Poland  Portugal  Spain  *Sweden*  Switzerland |
| **North Africa and Middle-East** | Egypt  *Iraq*  Jordan  *Lebanon*  Morocco  Tunisia  Turkey | *Israel*  *Qatar*  Saudi Arabia  United Arab Emirates (UAE) |
| **Sub-Saharan Africa** | Cabo Verde (Cape Verde)  *Ethiopia*  *Ghana*  Kenya  *Madagascar*  *Malawi*  *Rwanda*  South Africa  Tanzania  *Uganda* |  |

Countries with fewer than 4 visitors are indicated in italics.

Azores (Portugal), Ghana, Cambodia, Colombia, Nepal, Peru, Qatar, Singapore, Tajikistan, Vietnam, Afganistan, Antigua and Barbuda, Ethiopia, Iraq, Lebanon, Madagascar, Malawi, Malaysia, Maldives, Sri Lanka and Uganda had fever than 4 visitors but were only reported as destinations by cases.

**Supplementary Figure 1 – Epidemiological curve showing cases with a diagnosis of either Cryptosporidium, Giardia, non-typhoidal Salmonella and Shigella by week reported to UKHSA between week 27 and week 40 2023. Questionnaire respondents are shown in light grey and non-respondents in dark grey. The light grey horizontal line indicates the number of cases estimated to have travelled internationally based on previously published proportions.**


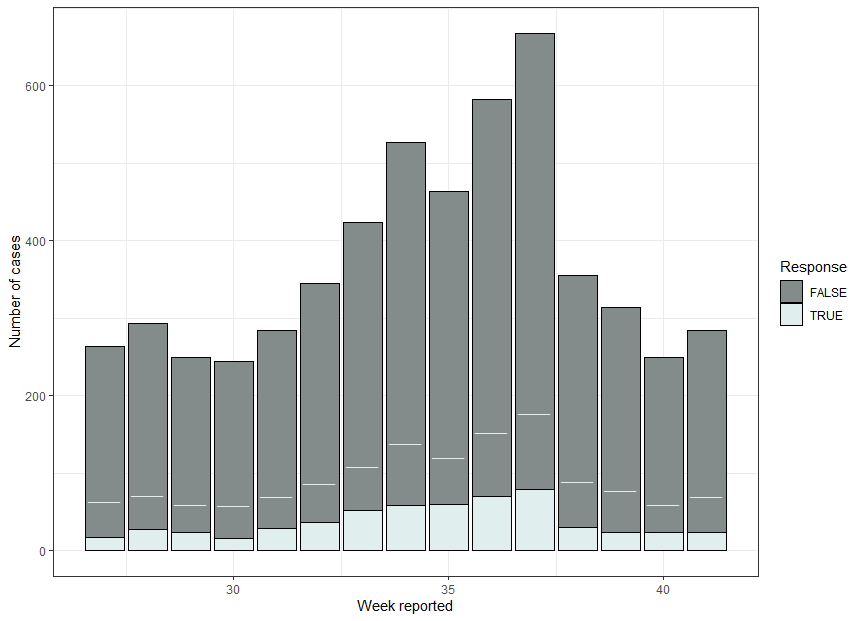


**Supplementary Table 2 - Location for seeking healthcare both while outside of the UK and within the UK. Respondents may have sought care from more than one location, both within UK and outside of UK. % denomination is total respondents reporting seeking care.**

|  | Outside UK | Within UK |
| --- | --- | --- |
| Location | N = 89^1^ | N = 653^1^ |
| Holiday Rep | 16 (18%) | - |
| GP | 25 (28%) | 542 (83%) |
| Pharmacy | 46 (52%) | - |
| Hospital | 12 (13%) | 87 (13%) |
| NHS111 | 1 (1.1%) | 153 (23%) |
| Online (non-NHS111) | 2 (2.2%) | 23 (3.5%) |
| Other | 13 (15%) | 20 (3.1%) |
| Walk-in centre | - | 32 (4.9%) |
| ^1^n (%) | | |

**Supplementary Table 3 – Self reported consumption of antibiotics and other medications both in the UK and while travelling**

|  |  | **Overall** | **Pathogen specific** | | | |
| --- | --- | --- | --- | --- | --- | --- |
|  | **Characteristic** | **N = 234^1^** | **Cryptosporidium, N = 15^1^** | **Giardia, N = 73^1^** | **Salmonella, N = 114^1^** | **Shigella, N = 21^1^** |
| **Antibiotics** | **Penicillin** | 20 (8.5%) | 3 (20.0%) | - | 13 (11.4%) | 4 (19.0%) |
|  | **Macrolides** | 18 (7.7%) | 1 (6.7%) | - | 15 (13.2%) | 1 (4.8%) |
|  | **Cephalosporins** | 5 (2.1%) | - | - | 4 (3.5%) | 1 (4.8%) |
|  | **Fluroquinolones** | 41 (17.5%) | 2 (13.3%) | 1 (1.4%) | 30 (26.3%) | 7(33.3%) |
|  | **Chloramphenicol** | 1 (0.4%) | - | - | 1 (0.9%) | - |
|  | **Trimethoprim and Sulfamethoxazole** | 1 (0.4%) | - | - | 1 (0.9%) | - |
|  | **Carbapenems** | 1 (0.4%) | - | - | - | 1 (4.8%) |
|  | **Metronidazole and Tinidazole** | 68 (29.1%) | 3 (20.0%) | 53 (72.6%) | 8 (7.0%) | 2 (9.5%) |
|  | **Rifamycin** | 3 (1.3%) | 1 (6.7%) | - | 2 (1.8%) | - |
|  | **Nitrofurans** | 2 (0.9%) | - | - | 1 (0.9%) | - |
|  | **Unknown** | 74 (31.6%) | 5 (33.3%) | 19 (26.0%) | 39 (34.2%) | 5 (23.8%) |
|  |  | **N = 653^1^** | **Cryptosporidium, N = 188^1^** | **Giardia, N = 83^1^** | **Salmonella, N = 308^1^** | **Shigella, N = 48^1^** |
| **Other medications** | **Antidiarrheals** | 211 (32.3%) | 41 (21.8%) | 34 (41.0%) | 107 (34.7%) | 20 (41.7%) |
|  | **Pain medication** | 334 (51.1%) | 102 (54.3%) | 20 (24.1%) | 180 (58.4%) | 23 (47.9%) |
|  | **Rehydration salts** | 250 (38.3%) | 74 (39.4%) | 30 (36.1%) | 125 (40.6%) | 15 (31.3%) |
|  | **Proton pump inhibitor** | 18 (2.8%) | 6 (3.2%) | 2 (2.4%) | 8 (2.6%) | - |
|  | **IBS relief** | 51 (7.8%) | 11 (5.9%) | 9 (10.8%) | 26 (8.4%) | 4 (8.3%) |
|  | **Anti-emetic** | 33 (5.1%) | 10 (5.3%) | 3 (3.6%) | 19 (6.2%) | 1 (2.1%) |
|  | **Probiotic** | 11 (1.7%) | 4 (2.1%) | 2 (2.4%) | 3 (1.0%) | - |
|  | **IV fluids** | 5 (0.8%) | 3 (1.6%) | - | 2 (0.6%) | - |
|  | **Herbal medication** | 15 (2.3%) | 7 (3.7%) | - | 6 (1.9%) | 2 (4.2%) |

^1^ n

**Supplementary Table 4 – Demographic charactertistics of cases (n=653) and controls (n=483) who responded to the electronic questionnaire**

|  | Variable | Controls  n = 483^1^ | Cases  n = 653^1^ | p-value^2^ |
| --- | --- | --- | --- | --- |
| Age group* | 0 - 9 years | 54 (11%) | 99 (16%) | <0.001 |
|  | 10 -19 years | 60 (12%) | 75 (12%) |  |
|  | 20 - 29 years | 36 (7.5%) | 84 (14%) |  |
|  | 30 - 39 years | 54 (11%) | 129 (21%) |  |
|  | 40 - 49 years | 72 (15%) | 91 (15%) |  |
|  | 50 - 59 years | 72 (15%) | 84 (14%) |  |
|  | 60 - 69 years | 77 (16%) | 39 (6.3%) |  |
|  | 70 years and over | 57 (12%) | 16 (2.6%) |  |
| Sex** | Female | 275 (57%) | 405 (63%) | 0.056 |
|  | Male | 207 (43%) | 241 (37%) |  |
| Ethnicity*** | Asian or Asian British | 22 (4.6%) | 30 (4.7%) | 0.40 |
|  | Black or Black British | 7 (1.5%) | 9 (1.4%) |  |
|  | Mixed: White and Asian | 7 (1.5%) | 6 (0.9%) |  |
|  | Mixed: White and Black | 13 (2.7%) | 13 (2.0%) |  |
|  | Other ethnic group | 11 (2.3%) | 11 (1.7%) |  |
|  | White - Other | 41 (8.5%) | 35 (5.5%) |  |
|  | White British | 379 (79%) | 535 (84%) |  |
| Duration resident in UK^#^ | 0 to 5 years | 8 (1.7%) | 11 (1.7%) | 0.003 |
|  | 16 years or more | 42 (8.8%) | 26 (4.0%) |  |
|  | 6 to 15 years | 29 (6.0%) | 25 (3.9%) |  |
|  | From birth | 401 (84%) | 581 (90%) |  |
| IMD decile^¥^ | 1 (most deprived) | 17 (7.6%) | 17 (5.0%) | 0.60 |
|  | 2 | 25 (11%) | 36 (11%) |  |
|  | 3 | 20 (9.0%) | 24 (7.1%) |  |
|  | 4 | 23 (10%) | 24 (7.1%) |  |
|  | 5 | 18 (8.1%) | 31 (9.1%) |  |
|  | 6 | 18 (11%) | 36 (11%) |  |
|  | 7 | 24 (7.6%) | 37 (11%) |  |
|  | 8 | 17 (7.6%) | 40 (12%) |  |
|  | 9 | 25 (11%) | 41 (12%) |  |
|  | 10 (least deprived) | 36 (16%) | 53 (16%) |  |
| Occupation | Food handler | 27 (5.6%) | 27 (4.1%) | 0.25 |
|  | Childcare/education setting | 29 (6.0%) | 36 (5.5%) | 0.72 |
|  | Health and Social care | 34 (7.0%) | 70 (11%) | 0.033 |
|  | Working with animals | 14 (2.9%) | 4 (0.6%) | 0.002 |
|  | Workplace with difficulties in maintaining hygiene | 8 (1.7%) | 4 (0.6%) | 0.089 |
|  | Any other occupation - outside home | 138 (29%) | 158 (24%) | 0.10 |
|  | Any other occupation – work from home | 59 (12%) | 98 (15%) | 0.18 |
|  | In education | 120 (25%) | 186 (28%) | 0.17 |
|  | Unemployed or retired | 98 (20%) | 105 (16%) | 0.067 |
| Comorbidities | Acid reflux | 78 (16%) | 88 (13%) | 0.21 |
|  | Inflammatory Bowel Disease | 0 (0%) | 16 (2.5%) | <0.001 |
|  | Diabetes | 28 (5.8%) | 22 (3.4%) | 0.049 |
|  | Immunosuppressed | 34 (7.0%) | 17 (2.6%) | <0.001 |
|  | Pregnant | 4 (0.8%) | 6 (0.9%) | >0.99 |
| Destination^≠^ | High-risk WASH destination | 61 (12.6%) | 288 (48.9%) | <0.001 |
|  | Low-risk WASH destination | 422 (87.4%) | 300 (51.0%) |  |
| ^1^n (%)  ^2^Pearson's Chi-squared test; Fisher's exact test  *Age missing or prefer not to say for 1 control and 36 cases.  **Sex missing or prefer not to say for 1 control and 7 cases.  ***Ethnicity missing or prefer not to say for 3 controls and 14 cases.  ^#^ Duration resident in UK missing for 11 controls and 21 cases.  ^¥^IMD (derived from postcode) missing for 260 controls and 314 cases.  ^≠^WASH score missing for 65 cases. | | | | |
